# Molecular Dating of HIV-1 Subtype C from Bangladesh

**DOI:** 10.1371/journal.pone.0079193

**Published:** 2013-11-01

**Authors:** Irene Bontell, Md. Safiullah Sarker, Mustafizur Rahman, Mokibul Hassan Afrad, Anders Sönnerborg, Tasnim Azim

**Affiliations:** 1 Unit of Infectious Diseases, Department of Medicine Huddinge, Karolinska Institutet, Solna, Sweden; 2 Virology Laboratory, icddr,b, Mohakhali, Dhaka, Bangladesh; 3 Division of Microbiology, Department of Laboratory Medicine, Karolinska Institutet, Solna, Sweden; University of Amsterdam, Netherlands

## Abstract

Bangladesh has an overall low HIV prevalence of <0.1% in the general population and <1% among key affected populations, but it is one of few Asian countries that has yet to reverse the epidemic. In order to do this, it is important to understand the transmission dynamics in this country. The aim of this study was to investigate the phylogenetic relationships of HIV-1 subtype C strains from Bangladesh and related strains from other countries, and thereby clarify when and from where subtype C was introduced in the country and how it subsequently spread within Bangladesh. The phylogenetic analysis included 118 Bangladeshi *gag* sequences and 128 sequences from other countries and was performed using the BEAST package. Our analysis revealed that the vast majority of Bangladeshi sequences (97/118, 82%) fall into a large regional cluster of samples from Bangladesh, India, China and Myanmar, which dates back to the early 1960’s. Following its establishment in the region, this strain has entered Bangladesh multiple times from around 1975 and onwards, but extensive in-country transmission could only be detected among drug users and not through sexual transmission. In addition, there have been multiple (at least ten) introductions of subtype C to Bangladesh from outside this region, but no extensive spread could be detected for any of these. Since many HIV-infections remain undetected while asymptomatic, the true extent of the transmission of each strain remains unknown, especially among hard to reach groups such as clients of sex workers and returning migrants with families.

## Introduction

Bangladesh is a low-prevalence country for HIV with an estimated overall prevalence of less than 0.1%. The prevalence in most of the key affected populations, such as female sex workers (FSW), men who have sex with men (MSM) and hijra/transgendered population (TG) remains below 1%, but the highest concentration of cases is found among people who inject drugs (PWID) in Dhaka, where the latest reported prevalence figure is 5.3% [1]. HIV-1 subtype C is highly predominant in this group and it is also the single most common subtype in Bangladesh [2]. The HIV-epidemics of several other countries in South and South-East Asia started off in PWIDs and then spread via needle sharing and unprotected sex to sex workers and thereafter to the general population [3]. Early intervention efforts in Bangladesh, including extensive needle-syringe exchange programs, are estimated to have reduced infections among PWIDs by as much as 90% [4] and also contributed to decreased transmission to other populations. However, the Bangladeshi HIV epidemic is still growing [5], with the majority of new infections detected among returning migrant workers [6]. Bangladesh is a major labour-sending country, mainly to the Middle East, with around 200,000-800,000 workers getting employed abroad each year over the past two decades [7]. Although going abroad to work is not a risk factor *per se* and the individual risk of contracting HIV remains low, this large group of people nevertheless contributes to a continuous in-flow of new HIV-infections to Bangladesh. In order to reverse the increasing trend and avoid the scenario experienced in harder-hit countries like neighboring states of India and Myanmar it is important to understand the transmission dynamics in this country. The aim of this study was to investigate the phylogenetic relationship between HIV-1 subtype C strains from Bangladesh and related strains from other countries, and thereby elucidate when and from where subtype C was introduced in the country and how it has subsequently spread within Bangladesh. 

## Materials and Methods

The analyzed viral sequences originated from blood samples obtained from serological surveillance surveys conducted by the International Centre for Diarrhoeal Disease Research, Bangladesh (icddr,b) on behalf of the Government of Bangladesh during 1999-2007, from research studies and from voluntary counseling and testing (VCT) units from 2002 to 2007. The sampling procedures, PCR and sequencing methodology have been described previously [2], and the majority of sequences had been submitted to GenBank previously (Accession numbers EF999759-EF999824, EU167547-EU167548, JQ668327-JQ668332, AF470702, AF470704, AF470707, AF470709, AF470710, AF448213-AF448217 and AF448219. New submissions for this paper: KC859271-KC859303). Informed and signed consent was obtained from all study participants prior to drawing blood, and in the case of children (N=5) consent was obtained from parents/guardians. The summary of the consent paper was read out for those who could not read and the left thumb impression was obtained from those who could not sign. This study, which included secondary analysis of already retrieved samples, was approved by the Ethical Review Committee of icddr,b. 

The age of the study population ranged from 2-59 years, 22% were female and 99/118 (83.8%) were from the capital, Dhaka. The population included 52 PWIDs, 2 heroin smokers, 10 FSW, 3 MSM, 2 TG and 49 people who were either VCT clients or were detected with HIV while seeking treatment for tuberculosis (TB, N=2) or other sexually transmitted infections (STIs, N=3). The VCT clients included five vertically infected children (ages 2-7), while the risk factor in the other cases was mostly unknown. However, 23 of the VCT clients had a history of travel to Saudi Arabia, Kuwait, United Arab Emirates, India, Nepal or Malaysia. 

A total of 118 Bangladeshi subtype C sequences were available for the *gag* gene. This gene is well suited for phylogenetic analysis as it is not as variable as *env* and not under selective pressure by antiretroviral drugs, like *pol*. For the phylogenetic analysis all sequences were truncated to start in the correct reading frame and cover exactly the same genetic region (corresponding to positions 934-1244 in the HXB2 reference strain). Reference sequences were retrieved from the LANL HIV sequence database [8] through BLAST searches for all included Bangladeshi strains (the five best hits were kept for each strain, duplicates were eliminated and only strains with known year and country of sampling were included). In addition, the geographic search interface was used to retrieve subtype C sequences from nearby countries, and 16 additional sequences from Myanmar were included. China and India were already represented from the BLAST search and subtype C sequences from other nearby countries including Nepal and Malaysia were not available. A search for sequences from the most popular destination countries for migrant workers (Saudi Arabia, UAE, Kuwait, Oman, Qatar, Bahrain and Libya) was also performed, but it was found that no subtype C sequences were available from this geographic region for the part of the *gag*-gene used in this analysis. Finally, subtype references for B, C, 07_BC and 08_BC were retrieved and included in the data set. The final set included sequences from Bangladesh (118), China (31), Myanmar (19), India (16), South Africa (14), Zambia (12),Malawi (9), Ethiopia (7), Israel (4), Botswana (4), Great Britain (2), Somalia (2), Zimbabwe (1), Denmark (1), Kenya (1), Tanzania (1), USA (1), Brazil (1), Thailand (1) and France (1). Alignments were performed in MEGA 5 [9], and manual editing was done to ensure that all strains were in the correct reading frame throughout the alignment. The final *gag* alignment included 246 taxa covering 374 sites. 

The phylogenetic analysis was performed using BEAST v.1.7.3 [10]. The GTR+Γ+I substitution model was used in all runs, since this was found by to be the most appropriate according to ModelTest [11]. Preliminary analysis included combinations of lognormal and exponential relaxed clocks with logistic growth and Bayesian Skyline tree priors. Bayes factor analysis performed in Tracer v.1.5 [10] showed that the lognormal relaxed clock with the Bayesian Skyline was the best model and this was used for the subsequent analyses. The final analysis consisted of two parallel runs of 20 million generations, with parameters logged every 1000 gen. Tip dates (year of sampling) were included for all sequences and four different taxons were defined: one with all subtype C strains, and three Bangladesh specific clades. The evolutionary rate for gag, 0.002 mutations/site/year, was used. Adjusted priors were the tree root height (mean 100 years, stdev 20) and a previously calculated tMRCA for subtype C (mean 58, stdev 6 [12]). The resulting log-files were analyzed in Tracer, while the final tree was compiled in TreeAnnotator using a burn-in of 1,000 trees and edited in FigTree [13].

## Results and Discussion

**Figure 1:**
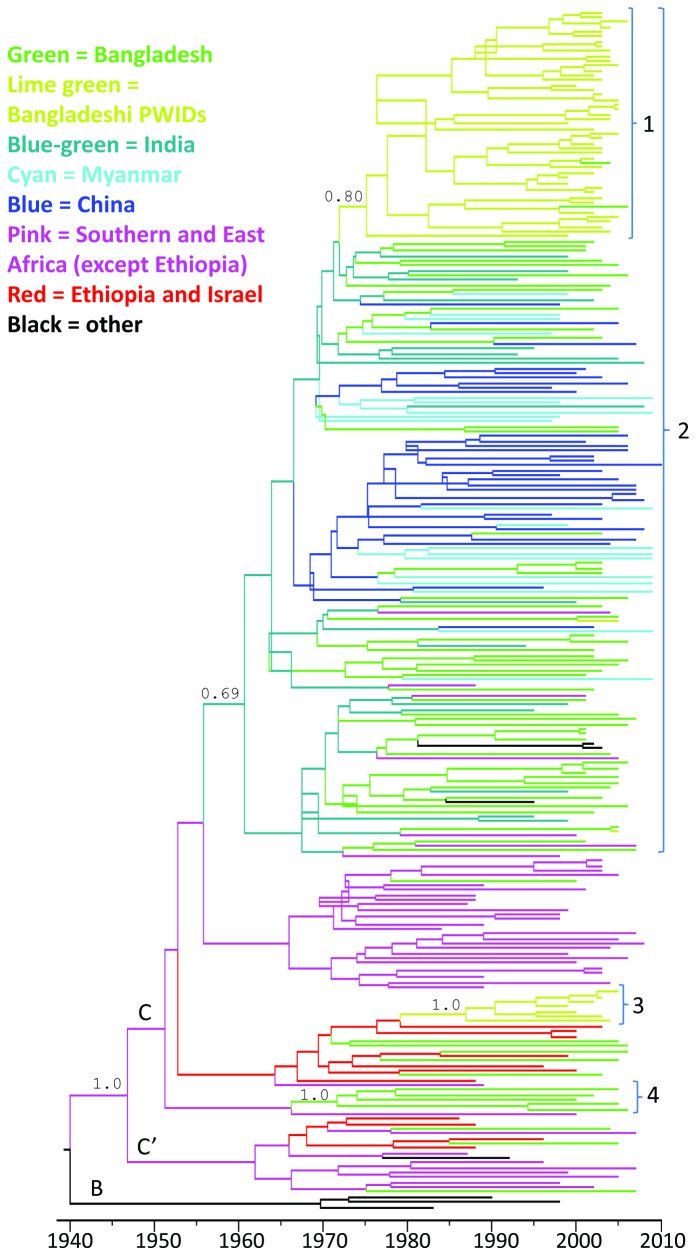
Phylogenetic tree of 246 *gag*-sequences, including 118 Bangladeshi HIV-1 C strains in green. The HIV1-C strains found in Bangladesh do not fall into a country specific clade, instead our results point to repeated introductions with substantial in-country transmission only among PWIDs. Four clades with ≥3 BD strains had a posterior probability of around 0.7 or higher, and are shown in the figure. ***Clade 1*** is a monophyletic clade of 47 BD strains, of which 43 originate from PWIDs in Dhaka. The tMRCA of this clade is around 1975. It is a subset of the large regional ***Clade 2***, which is dominated by strains from Bangladesh, Myanmar, India and China. Over 80% of all BD strains can be found within this clade, which appears to have been introduced to this region around 1962. The intermixing of sequences collected from different countries indicates frequent cross-border transmission in this region. In addition to the 47 strains in Clade 1, the BD strains in Clade 2 includes sequences from returning migrants from Nepal, India and the Middle East as well as some weakly supported clades of strains from sex workers and VCT/STI patients. ***Clade 3*** consists of seven PWID strains from Dhaka, and the short branches in this clade reveal rapid transmission (and detection) within this group. ***Clade 4*** contains five strains from VCT visitors in Dhaka and Chittagong, at least 3 of whom had a history of migrant work in the Middle East. The long branches show that they are not very close genetically and it is possible that they represent separate introductions of related strains. Most of the BD strains found outside these clades appear to represent separate introductions.

Our analysis of 118 HIV subtype C strains from Bangladesh reveal that the vast majority of Bangladeshi strains (97/118, 82%) fall into a large cluster of HIV strains sampled mainly from Bangladesh (BD), India (IN), China (CN) and Myanmar (MM), (Figure 1). There has been repeated transmission between Bangladesh and these three nearby countries, and many BD strains are intermixed with strains from IN, CN and MM. The combined calculated tMRCA of this clade for the two BEAST runs was the year 1962 (95% CI: 1951-1969). However, the first introductions into Bangladesh occurred around 1975, which is 14 years before the first known HIV-case in the country and over two decades prior to the first identification of subtype C in Bangladesh in 1997 [14]. The ancestral strain for this south Asian cluster appears to originate from southern Africa, but the exact country could not be determined. Six of the BD strains located outside the main clade were also genetically related to strains from southern Africa (especially Zambia), while twelve BD strains were more closely related to strains from Israel and Ethiopia. Furthermore, three strains from Bangladesh could be classified as sub-subtype C’, which is common in Ethiopia. These three strains were not closely related and originated from seemingly unrelated male VCT visitors who were all returning migrant workers (one from Malaysia and two from Saudi Arabia) and most likely represent separate introductions. 

The highest HIV prevalence in Bangladesh is found among PWID in Dhaka, and subtype C is common among these patients. Within the major BD/IN/CN/MM cluster there was a large monophyletic clade of 47 strains collected in Dhaka. One of these 47 strains originated from a TB patient and one from a VCT client, but the overwhelming majority were collected from people who use drugs (2 heroin smokers and 43 PWID). The tMRCA of this clade was calculated to around 1975 (95% CI: 1969-1981), which is twenty-four years prior to the first known cases of HIV among PWID in 1999 [15]. According to our phylogenetic analysis, the Bangladeshi PWID strains are closely related to PWID strains from Myanmar. Drug trade across this border is common and due to political unrest many people from Myanmar have fled to neighbouring countries including Bangladesh [16], so transmission via this route is highly plausible. 

A separate, genetically unrelated cluster of strains from seven PWIDs from Dhaka was found outside the main clade. Their tMRCA was found to be around 1987 and the ancestral strain appears to come from Israel or Ethiopia. Analysis of patient travel histories revealed that the route of introduction to Bangladesh was most likely through migrant workers who got infected in Saudi Arabia, and then this strain somehow entered the PWID population, where it got disseminated further. A small number of strains from PWIDs in Bangladesh were found outside these two clusters; one was highly similar to a strain from a FSW from Hili (a small town in the northwest, on the border to India) and another one clustered tightly with a strain from a VCT patient in Chittagong (a port city in the southwest). In both cases, transmission appears to have been recent as the genetic similarity is very high, and no further transmission within the PWID community appeared to have taken place at the time of sample collection. Additional PWID strains were identified from a 34 year old male with frequent trips to India, which clustered with a strain from a male PWID from Myanmar, and from a 31 year old male with travel history to Nepal. Risky injecting and sexual behaviour among PWID in this region have been reported previously [17] and a two-way flow of HIV across the Indo-Nepal border through injecting and sexual networking has been established through analysis of serological and behavioural data from a south-eastern cluster of Nepal and a north-eastern district of India [18]. A high genetic similarity between strains sampled in different countries is therefore expected.

Through careful examination of all available patient data, several additional cases of plausible acquisition of HIV-C abroad were detected. At least three strains appears to have been acquired through heterosexual commercial sex by Bangladeshi men travelling in India; one was detected in a 30-year old male who bought sex in India and the other two were detected in young women whose spouses admitted to have visited sex workers in India. This type of transmission between husband and wife often turns out to be a cul-de-sac for the virus, but there is a risk that the infection, unless detected in time and properly treated, is transmitted to future children. One Bangladeshi FSW had previously worked in a Mumbai brothel and it is likely that she acquired the strain there. There is no evidence that this strain has been further transmitted within Bangladesh, but as the latent period can last many years there is a risk that clients of this sex worker and their partners carry undetected infections. 

In addition, at least seven Bangladeshi strains appear to have been introduced by returning migrants from the Middle East (four male and one female from Saudi Arabia and two male from Kuwait). Genetically, these strains clustered with African strains and three of them belonged to a small monophyletic cluster of five strains sampled from VCTs and STI patients in Dhaka and Chittagong with a tMRCA around 1972. The closest strain outside this cluster was isolated in Zambia in 2000, but it has been reported previously that many African strains were imported to Saudi Arabia due to close geographical proximity and commercial interaction [19]. Bangladesh provides a strong labor market to the Middle East, especially Saudi Arabia, and it seems plausible that with a lack of strong ties to countries in Southern and East Africa, the African strains now circulating in Bangladesh have been introduced by migrant workers spending time in Saudi Arabia and other Middle Eastern countries. It is well established that returning migrants constitute a major proportion of the total annual detected HIV cases in Bangladesh, and these strains belong to a wide variety of subtypes and recombinant forms [20]. Unfortunately, no subtype C sequences isolated in the Middle East were available for the examined part of the *gag*-gene, and it was thus not possible to determine whether this cluster of five reflects transmission within Bangladesh or if the infections were acquired independently by migrant workers in the Middle East. 

Our study has a number of limitations, especially that the most recent sequences from Bangladesh were sampled in 2007. Inclusion of samples from later years would be needed for a more accurate assessment of transmission patterns. The sample size of 118 is also limited, but actually corresponds to nearly 10% of the 1207 detected HIV cases in Bangladesh by 2007, and can therefore be seen as fairly representative of the known epidemic. Due to the huge population and low prevalence in the country, surveillance has been limited to key affected populations, and therefore transmission outside these groups may largely remain undetected until symptomatic. The size of the alignment is only 374 bp and the analysis would be strengthened by the use of longer sequences and/or inclusion of multiple genes, but only a very small number of sequences were available for other genetic regions. However, the main clades described in the tree have posterior probability supports around 0.7 or higher and therefore the overall phylogeny is fairly robust although some questions remain regarding the finer details. 

In summary, most of the detected HIV subtype C cases in Bangladesh belong to one large regional cluster with strains from India, China and Myanmar. Cross-border transmission appears to be very common in the region, while extensive HIV transmission within Bangladesh was only detected among people who inject drugs, and not for sexual transmission. Two genetically distinct strains of subtype C are circulating among PWID in Dhaka, and the larger clade contained nearly 40% of the Bangladeshi strains included in the study. In-country sexual transmission between small numbers of individuals (2,3) was repeatedly observed, but the larger clusters of sex workers and STI or VCT patients had low posterior probabilities and the possibility of multiple introductions of genetically related strains could not be excluded. In total, we were able to establish at least ten separate introductions of HIV-1 subtype C to Bangladesh from various locations, but this figure is probably much higher as cross-border transmission through intravenous drug use and commercial sex appears to be very common in the region. Since most HIV-infections remain undetected while asymptomatic, the true extent of the transmission of each strain remains unknown, especially among hard to reach groups such as clients of sex workers and returning migrants with families. Regular surveillance of key affected populations in Bangladesh has resulted in earlier detection especially among PWIDs in Dhaka, as seen by the short branches in the tree. Early targeted interventions among key affected populations in Bangladesh have most likely contributed to keep the national HIV prevalence at a very low level. However, our results demonstrate a steady introduction of new strains from various countries, as well as repeated cross-border transmission with neighboring states and expanded interventions for earlier detection and treatment of HIV among returning migrants and their families should be a priority in order to contain and reverse the HIV epidemic in Bangladesh.
